# Neural Network Based IRSs-UEs Association and IRSs Optimal Placement in Multi IRSs Aided Wireless System

**DOI:** 10.3390/s22145216

**Published:** 2022-07-12

**Authors:** Ahmed M. Nor, Simona Halunga, Octavian Fratu

**Affiliations:** 1 Department of Telecommunications, University Politehnica of Bucharest, 060042 Bucharest, Romania; simona.halunga@upb.ro (S.H.); octavian.fratu@upb.ro (O.F.); 2 Department of Electrical Engineering, Aswan University, Aswan 81528, Egypt

**Keywords:** intelligent reflecting surfaces (IRS), neural network, IRSs-UEs association, passive beamforming, optimal placement, 5G and beyond networks

## Abstract

Implementing intelligent reflecting surfaces (IRSs), in high frequency based beyond 5G networks, has become a necessity to overcome the harsh blockage issues that exist in these bands. IRSs can supply user equipment (UEs) with multi alternative virtual line of sight (LOS) links, hence enhancing the spectral efficiency (SE) of the system. As a result of deploying multi IRSs as communication assistants, the step of IRSs-UEs association is required to optimally assign each UE to its best IRS; consideration of the interference between different links is needed, to maximize the system performance. However, this process will be a time and power consuming problem, if conventional schemes, which exhaustively search all possible association patterns to find the optimum one for communication, is adapted. Although iterative search based schemes can reduce this complexity, they still need feedback signaling in real time. Hence, they will be inefficient in terms of power consumption and delay. Moreover, optimal placement of the multi-IRSs in the network, to enlarge the system performance, is still an open issue and needs to be studied. Consequently, in this paper, to handle the IRSs-UEs association problem, we propose a neural network (NN) based scheme using a multi-IRSs aided multi input multi output (MIMO) system. In this system, the estimated angles of arrival (AoAs) of UEs are used as input features for the NN, which is trained to associate each UE to its best IRS based on this information; then, within each IRS, passive beamforming is performed. Adapting this NN in online mode guarantees obtaining better performance while relaxing the complexity of association and increasing response time, giving a performance comparable to the exhaustive and iterative search based schemes. The proposed NN based scheme determines the association pattern without searching or feedback signals. Moreover, the proposed approach maintains the system SE nearly similar to the optimum performance obtained by the conventional scheme. Secondly, a criterion is suggested for optimal deployment of multi IRSs in the network, depending on maximizing the average summation UEs signal-to-interference-plus-noise ratio (SINR). Numerical results prove that this strategy outperforms a reference one, which aims to guarantee certain performance by maximizing minimum UE SINR. In contrast the proposed strategy achieves better system and per UE spectral efficiency.

## 1. Introduction

Recently, the demand for ultra-high data rate, ultra-low latency, wide coverage and connectivity, and high reliability and energy efficiency networks has increased [1,2,3]. For instance, according to annual internet report from Cisco [4], by the end of 2023, the global average Wi-Fi speed will increase to 92 Mbps, while machine to machine connections, which are thirsty consumers for low latency real-time communication, will total 14.7 billion. Hence, much attention is being given to research on 5G and beyond networks. These networks can be enabled by several solutions such migration to high frequency bands, e.g., millimeter wave (mmWave) [5,6] and terahertz (THz) bands [7,8]. Although these bands, that work in GHz spectrum, are promising candidates for 5G and 6G networks as they can handle the former demands, they have some limitations, which diminish their performance and efficiency. Firstly, propagation losses increase greatly on high frequency bands in this range of GHz, following Friis’s law, decreasing their coverage and limiting their applications [6]. To solve this, antenna arrays with beamforming abilities are adopted for mmWave and sub-terahertz transmitter (TX) and receiver (RX) [9]. Second, high frequency bands are highly susceptible to blockage from static and dynamic obstacles due to poor penetrability, i.e., obstacle barriers strongly attenuate the mmWave and THz signal, and thus direct line of sight (LOS) between TX and RX can be lost, leaving only weak non-line of sight (NLOS) links for communication [10,11,12]. Consequently, a new promising solution based on intelligent reflecting surfaces (IRS) can be considered to overcome the blockage issue in beyond 5G networks [13].

IRS is a planar flat surface which contains a large number of passive reflecting meta elements. These elements can be controlled to redirect the incoming signal from the TX to certain locations where RX exists [14], after adjusting the amplitudes and phase shifts of these elements. Deploying IRSs in beyond 5G networks to assist in communication plays a vital role in enhancing the network performance by providing alternative indirect virtual LOS links between TXs and RXs when the main links are blocked by obstacles. These links can not only be used for communication, but also can be beneficial for positioning services, mapping, and sensing [13,14]. Furthermore, implementing IRSs can expand the network coverage, increase the channel rank, and refine its statistics. Unfortunately, all these benefits do not come alone, as new challenges appear in IRS assisted wireless systems. First, the phase shifts design of passive reflecting elements is required at each IRS in the system to optimally direct the incident signal to the target RX, which is called a passive beamforming (P-BF) process [15]. In addition, joint optimization of P-BF and multi input multi output (MIMO) transmit covariance matrices [16], is required to optimally establish communication links. Moreover, to serve several pieces of user equipment (UEs) in multi IRSs aided MIMO system in a downlink scenario, which is the main concern of our paper, IRSs-UEs association is needed to efficiently make use of network resources. This process objective assigns each UE to its best IRS in the network to guarantee maximizing total signal-to-interference-plus-noise ratio (SINR). However, the assignment problem requires searching time and power to find the optimal association pattern in real-time, so a solution to it is proposed here. In addition, the placement of multi IRSs in the network, which is far away from active relays deployment, impacts on the realization of IRSs-reflected channels, and as a result on the overall performance of the network [14,17]. Thus, investigating the optimal placement of IRSs with consideration of the mutual interference between the IRSs-UEs links is mandatory due to their high effect. However, other factors such as deployment cost, UEs distribution, and space constraints, should also be considered to maximize the performance of the network.

The concept of functional split between the control (C) plane and the user (U) plane has been widely adopted for 5G networks in heterogenous network (HetNet) paradigm [18,19,20,21,22], where controlling signals can be guaranteed by a low frequency band due to its wide and robust coverage, e.g., Wi-Fi band, while communication is provided using mmWave band. This HetNet architecture can still be considered the ideal candidate for indoors beyond 5G networks deployment, because a sub 6 GHz band can be used to relax some issues facing multi IRS aided wireless communication systems such as active-passive BF process and IRSs-UEs assignment problems. Based on UE context information (CI) provided using a Wi-Fi band, e.g., angle of arrival (AoA), positioning, application requirements, and expected mobility, the system can efficiently assign certain UE to certain IRS when more than one is available in the network. In addition, design of P-BF can be sub-optimally performed depending on the estimated UE position. Horizontal handover for UEs can be done based on the expectation of users’ mobility.

Recently, artificial intelligent (AI) techniques have been widely employed in wireless communication system based on users CI for several problems, e.g., association [19], beam management [21,23], spectrum management, resource allocation [24], and user positioning [25], because of their power in solving complicated problems in an energy efficient manner. For example, neural networks (NNs) have used sub-6 GHz measurements as input features to determine the best beam pairs for mmWave transmission [11]. Consequently, a NN approach can remain as a protentional solution to solve energy consumption searching processes that are required in IRSs aided MIMO system, where it can learn the optimal IRSs-UEs assignment pattern from previous knowledge. In addition, the beam management issue, that is challenging especially for a multi IRSs aided MIMO system, can be solved by the means of NNs, where prediction of IRS phase shifts can be made with the assistance of environmental awareness and user context information. In general, using machine learning based schemes over other methods can guarantee achieving superior performance with low complexity, fast response, high robustness and less human effort. For example, adapting exhaustive or iterative search based schemes to solve optimization problem for IRSs-UEs association, needs high power consumption and wastes a long time in real time, which means additional delays and power requirements. Furthermore, with higher numbers of IRSs and UEs, the complexity of obtaining the optimum IRSs-UEs pattern will increase. Moreover, trying fuzzy logic based schemes will not be efficient, because they require too many inputs and rules to achieve a good performance. In addition, to design statistical based models, intensive mathematics to estimate different coefficients, a good understanding of data, and robust assumptions based on probability space are required. However, this will not confirm obtaining acceptable performance for the network because the wireless communication environment has several parameters and changes from scenario to another.

Motivated by the aforementioned discussion and the superiority of using available CI from Wi-Fi band as input features for NNs, the contribution of our work in this paper can be summarized as follows:

- First, we introduce an optimization problem (OP) to jointly solve IRSs-UEs association and P-PF design problems. This OP aims to maximize the total SINR to obtain better system performance in terms of spectral efficiency. Mutual interference between different virtual links is considered. Then, due to this OP complexity, we propose dividing it into two stages. First, for the IRSs-UEs assignment problem, a neural network pattern recognition scheme based on out-of-band information is proposed for the first time in such a problem, to best of our knowledge. In this scheme, estimated UEs angle of arrival (AoA) data, that can be generated from Wi-Fi band using multiple signal classification (MUSIC) algorithm, are used as input features for our NN. Secondly, to design the IRSs phase shift, we adapt customized channel estimation based P-BF scheme that had been proposed in [26].
- Furthermore, we discuss optimal placement of multi-IRSs in a MIMO system, where our placement criterion aims to maximize the average summation of UEs SINR in the network. We assume that two IRSs will be implemented in the network. Hence, to find out the optimum positions for this pair of IRSs, we will search forall possible positions for this pair. This step is performed once before implementing the IRSs in the network during the process of designing the network.
- Finally, numerical results prove that the proposed NN based IRSs-UEs assignment scheme can achieve nearly the same performance obtained by the optimum exhaustive search based solution in terms of system spectral efficiency, while reducing the searching complexity, and hence consumed power, with 50% and 44% comparable to the exhaustive and iterative search based scheme. In addition, even if TX antenna gain or vertical distance between IRSs and UEs is changed, the proposed scheme can obtain similar performance to the conventional exhaustive search scheme. Furthermore, our results demonstrate that the proposed multi IRSs deployment criterion obtains better total system SE, comparable to the reference deployment strategy that was suggested in [27]. In addition, a larger data rate can be provided to each UE.

The reminder of the paper is organized as follows: Section 2 presents related works, that discuss AP-IRSs-UEs association, or that studied the optimal placement of IRSs in 5G networks. In Section 3, the multi IRSs aided MIMO network architecture, IRS configuration, and the link model for communication band are explained. Section 4 introduces the problem formulation for IRSs-UEs association and describes how we can solve it. The criterion for optimal IRSs placement in the network is also discussed. In Section 5, the proposed NN based scheme for IRSs-UEs association is presented in detail. Section 6 illustrates the simulation results for evaluating the proposed association scheme and optimal placement strategy. Finally, Section 7 concludes the paper.

## 2. Related Work

In this section, we will review the studies that have discussed IRSs-UEs association problem in multi IRSs aided MIMO systems. Then, we will overview the works that have explored the problem of optimal placement of IRS in wireless communication systems.

For IRSs-UEs association problem, few works in the literature discuss it in detail. The author in [28] proposed optimizing IRSs-UEs associations to maximize the minimum average-signal-to-average interference-plus-noise ratio (ASAINR). This OP can be solved based on exhaustive search of all association patterns, but it will be complicated in practice, especially for large numbers of IRSs. Hence, the authors proposed solving it using branch-and-bound (BB) method to relax its complexity. However, the power and time consumption required are still under question, because this iterative switching based scheme is performed in real time and needs feedback signaling at each iteration to converge. Thus, it produces additional delay to the system and consumes power due to passive BF which is performed for each IRSs-UEs association pattern. The authors in [29] proposed a dynamically successive access algorithm to obtain the APs-UEs association, where single IRS is implemented in the network which limits the superiority of multi IRSs deployment. Moreover, the authors in [30] used instantaneous channel state information (CSI) to design the user association (UA) and P-BF. In [31], the authors formulated an OP for a UA problem in an IRS aided mmWave network and solved it using a matching game assuming the availability of CSI in the base station and UE. However, to use CSI, it is necessary to estimate it in a previous step which is difficult and needs high overheads. In addition, in these studies [29,30,31], a single IRS is assumed to assist each base station, which is not the case in our scenario. Moreover, in [32], the authors proposed a scheme, which adapts priority based swapping and fixed point iteration algorithms for joint design of UA and P-BF in an IRS aided heterogenous network, respectively; statistical CSI is used to maximize the weighted sum rate of the network. However, this work considered the case where there is no mutual interference between reflected links from same band IRSs. Furthermore, some related works had considered multi IRSs aided wireless communication such as [15,16,33,34]. All these studies ignore optimum IRSs-UEs association and the mutual interference that happens between IRSs-UEs links, assuming association is done and given to the system.

Recently, optimal placement of IRSs occupies a big space in beyond 5G research [17,27,35,36,37,38,39]. In [35], an optimization, based on the IRS orientation and the distance between IRS and AP, was proposed to extend the IRS coverage. Authors in [36] proposed joint optimization of the IRS placement and its phase shifts to maximize the worst scenario signal-to-noise ratio (SNR) in air to ground 6G network. Works in [17,37] discussed the IRS deployment problem aiming to enhance the SNR level and diminish the blockage probability between the TX and the RX in mmWave network, respectively. In [38], considering adjacent cells interference, authors studied the optimal placement of IRS to widen the coverage and improve the performance of the network. The authors in [39] discussed the optimal IRS placement problem in a multiusers scenario, in which they proposed weighted sum rate maximization to jointly optimize the IRS position and its phase shifts, thus proving that the optimal placement of single IRS has a vital impact on the performance of the network. In [27], the authors discussed the deployment of an IRS based on guaranteeing minimum received power at UE in an indoor environment, considering different transmitter gains and mobile UE in the study area. Unfortunately, all these studies considered the scenario with single IRS in the network where no interference signal, generated from other IRSs, affects the received signal at UE.

## 3. System Model

**Figure 1:**
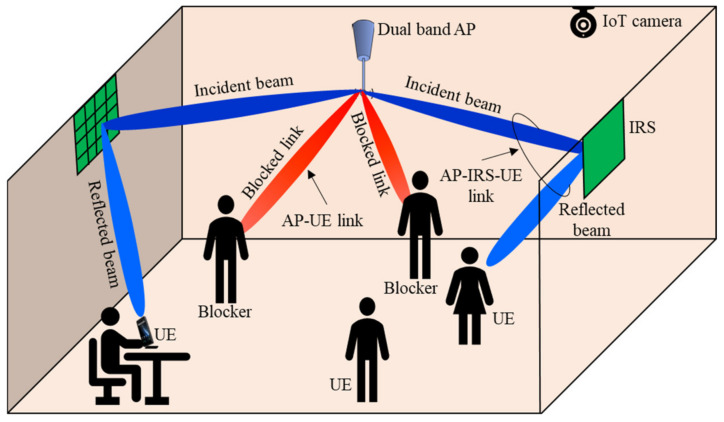
The network architecture of multi IRS aided MIMO communication system.

The network architecture of our multi IRSs aided MIMO system in a downlink scenario is presented in Figure 1. Here, we adopt a control/user (C/U) plane splitting concept, where a Wi-Fi band, i.e., 5 GHz, is used as a C plane for exchanging information between AP and UEs and sending control signals to network devices, due to its omnidirectional propagation, wide coverage, and reliability against blockage. On contrast, a sub THz band, i.e., 150 GHz, is used as a U plane for communication purposes due to its wide bandwidth and low latency. Hence, we assume dual band AP and dual interface UE, which can work in 5 GHz and 150 GHz bands. Moreover, all operations needed in this network, e.g., association, assignment, and passive BF design, are performed in AP side mode to save limited UE resources. The network consists of M antennas AP and K UEs with a single antenna, which communicate with each other through L passive IRSs by establishing indirect AP-IRSs-UEs links. The dual band AP is hinged on the room ceiling while IRSs can be placed on the walls due to their flat surfaces. The study area is a medium size indoor area, e.g., a medium office in a company. The direct AP-UEs links are represented by a red beam. We assume that direct links are fully blocked, while the indirect links between AP and UEs are exemplified by the blue color. These links are established using the help of IRSs, where they reflect the beams coming from AP to their assigned UEs based on their passive beamforming capabilities, i.e., IRSs elements are adjusted in phase and amplitude to redirect the incoming beams to UEs. The dark blue beams indicate to the AP-IRSs links, while light blue beams refer to the links between IRSs-UEs. We assume that each IRS l can be associated with only one UE k. Fiber optics links connect all network components.

**Figure 2:**
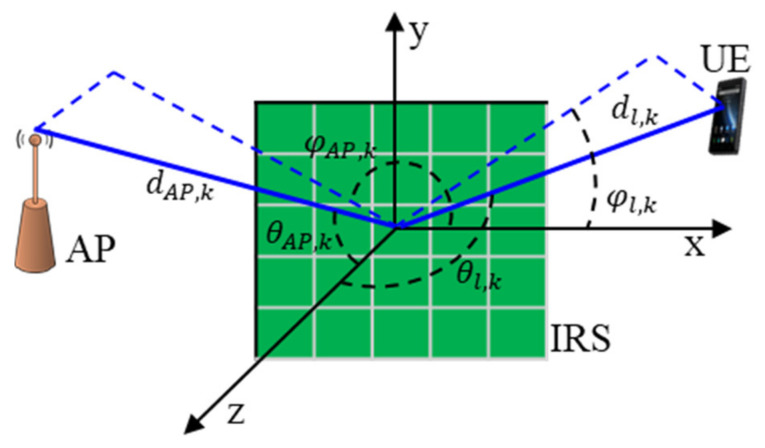
The configuration of intelligent reflecting surfaces (IRS).

Each l IRS consists of N passive elements and a control unit, that is responsible to adjust phase shift of each element. This unit can be a micro-controller that can get its orders and decision from the AP. To overcome blockage between AP and UE, the IRS reflects the incident signal coming from the AP and directs it towards the UE, after setting the optimum phase shifts of its elements, i.e., to perform passive BF for lth IRS. Performing P-BF is the process of determining $\Theta =diag\left\{\vartheta \right\}$, which is the diagonal phase shift matrix of the IRS, here $\vartheta ={\left[\vartheta_{1},\;\vartheta_{2},\ldots ,\;\vartheta_{N}\right]}^{T}$ and $\vartheta_{n}=\gamma e^{j\phi_{n}}$. The $\phi_{n}\in \left[0,2\pi \right]$ is the phase shift of IRS reflecting element n, and $\gamma \in \left[0,1\right]$ is the reflection coefficient amplitude. Several works have been proposed in the literature to perform P-BF using channel estimation, while the estimation of channel coefficients for AP-IRS-UE links in IRSs aided MIMO communication system is discussed in detail [26,40,41]. However, this is not the main scope of this work so we use the model described in [26] to perform P-BF. Then, we consider IRS l as one regime following the work in [42] to calculate the received power at UE coming from AP by the assistance of IRS. The configuration of IRS is presented in Figure 2, where the IRS is centered in the origin of coordinates system and IRS elements are represented as a green-colored rectangular shapes, which are distributed along x and y directions. The distance between AP and IRS center is $d_{AP,k}$ while the elevation and azimuth angles seen by IRS are $\theta_{AP,k}$ and $\varphi_{AP,k}$, respectively. The distance between UE and IRS center is $d_{l,k}$ while the elevation and azimuth angles seen by IRS are $\theta_{l,k}$ and $\varphi_{l,k}$, respectively.

We follow the model proposed in [42], assuming 150 GHz carrier frequency and only the existence of LOS paths, to describe the indirect LOS links between AP and UEs through IRSs, where the received power $P_{r}$ at UE *k* from the link reflected by IRS *l*, can be expressed as [42]:

(1) $$P_{r}=A_{r}S_{r}{}_{l,k}$$

where $A_{r}$ is UE effective aperture and presented as:

(2) $$A_{r}=\frac{G_{r}\lambda^{2}}{4\pi }$$

and where $G_{r}$ indicates to UE antenna gain and λ is the wavelength of free space. While, $S_{r}{}_{l,k}$ is the power density at UE *k*, which can be expressed as [42]:

(3) $$S_{r}{}_{l,k}=\frac{\frac{2P_{t}}{\lambda Z_{R}}{\left|R\right|}^{2}}{\sqrt{\left(1+\frac{d_{l,k}{}^{2}}{Z_{R}{}^{2}}\right)\left(1+\frac{d_{l,k}{}^{2}}{Z_{R}{}^{2}{cos}^{4}\theta_{l,k}}\right)}}$$

where $P_{t}$ is the AP transmitted power, and $\left|R\right|$ refers to the common reflection amplitude of all elements of IRS while $Z_{R}$ is the Rayleigh length and can be written as:

(4) $$Z_{R}=\frac{4k_{o}d_{AP,k}{}^{2}}{G_{t}}$$

where $k_{o}$ is the free space wavenumber and $G_{t}$ indicates to the antenna gain of AP. Hence, the signal to interference plus noise ratio at UE *k*, coming from AP through IRS *l*, can be expressed as:

(5) $$SINR_{l,k}=\frac{A_{r}S_{r}{}_{l,k}}{\sum_{i=1,\;\;i\neq l}^{L}A_{r}S_{r}{}_{i,k}+\sigma_{o}{}^{2}}$$

where $\sigma_{o}{}^{2}$ is the standard deviation of additive white gaussian noise at the *k*th UE.

## 4. IRSs-UEs Association and Optimal Placement of Multi IRSs

### 4.1. IRSs-UEs Association in Multi IRSs Aided MIMO System

The scenarios without interference, i.e., single user and single IRS cases, need only a P-BF optimization problem to design the phase shifts of IRS elements. In contrast, in a multi IRSs service multi-users scenario such as ours, *K* UEs need to connect to the AP through *L* IRSs. Hence, to establish the optimum AP-IRSs-UEs communication links for all possible configurations of IRSs-UEs links should be examined, and for each IRS-UE link, P-BF process should be performed. This process can be defined as a joint IRSs-UEs association and passive BF optimization problem (OP). The aim of this problem is to select the optimum IRS with its optimum elements’ phase shifts to serve certain UE. The mutual interference between IRSs-UEs links plays a critical role as it effects on the overall performance of the network, because the received signal at a certain UE, from its assigned IRS, is affected by the signal coming from other IRSs in the network. Hence, this joint OP should be based on SINR of UEs. Consequently, the target cost function of this problem can be formulated to maximize the total UEs SINR, as:

(6) $$\underset{w,\vartheta_{n}}{max}\overset{K}{\underset{k=1}{\sum }}SINR_{l,k},\;\mathrm{Subject}\;\mathrm{to}\;\sum_{k\in K}w_{l,k}=1,\;\forall l\in L,\;\left|\vartheta_{n}\right|=1,\;\forall n\in {Ι}_{N}$$

where $w=\left(w_{l,k}:l\in L,k\in K\right)$ is the indicator matrix for the IRSs-UEs assignment with size L×K. Here, $\vartheta_{n}=\gamma e^{j\phi_{n}}$ as described in Section 3, and ${Ι}_{N}$ is the set of integers from 1 to N.

To solve this problem, exhaustive search (ES) over all possible configurations can be performed, and, within each pattern, designing of IRSs elements’ phase shifts is performed using available methods in the literature for single IRS assisted MIMO systems. So, we will assume ES for IRSs-UEs association and the proposed P-BF scheme in [26] as the conventional scheme for comparison purposes. The addition of IRSs-UEs assignment to the already complicated P-PF process makes the overall process waste more power and time to find the optimum phase shifts of IRSs elements for each available assignment pattern. Simply, the overall complexity is the multiplication of the number of configuration patterns, which equals to $K!/\left(K-L\right)!$, by the time or power consumed to perform active passive PF for AP-IRS-UE link. Hence, to relax this complicated and time consuming process, we divide this OP into two separated stages, firstly, the IRSs-UEs association problem, which we propose to solve using neural networks as we will discuss in the next section, and the P-BF problem, for which we will adapt the scheme in [26]; other schemes might be considered, but this is outside the scope of this paper. It is worth mentioning that the complexity of this P-BF scheme, when it is implemented in our scenario, is $O\left(2QM+Q\left(M^{3}+M+1\right)\right)$, where Q is the number of training periods. Here, Q is assumed to be 10, and M=4. Furthermore, for more clarification of the obtained performance of our proposal, we assume a random association based scheme, which simply associates UEs to IRSs randomly without searching for the best IRSs-UEs pattern. Although the complexity of this scheme is similar to our proposed NN based scheme, its performance is much lower, as we will discuss.

### 4.2. Optimal Placement of Multi IRSs in MIMO System

Obtaining optimum performance needs optimal deployment of IRSs in the network to maximize the total rate. Thus, in this subsection, we will discuss the idea of optimal placement of the multi IRSs. Based on the concepts presented in the previous subsection, to optimally deploy multi IRSs, the mutual interference between IRSs-UEs links in the network should be minimized. Thus, we will optimally solve OP in (6) using an exhaustive search scheme to evaluate the optimum performance of each possible position for the multi IRSs. Our placement method is to select the optimal positions of the IRSs, where the average summation of UEs SINR is maximized. This criterion can be written as:

(7) $$maximize\bar{\overset{K}{\underset{k=1}{\sum }}SINR_{l,k}},$$

To find the optimum positions using (7), a searching for all possible positions for the IRSs as the function of UE positions with all possible configurations is performed. This criterion guarantees acceptable performance in terms of average total spectral efficiency (SE) of UEs and SE per UE. Moreover, it can achieve $\bar{\overset{K}{\underset{k=1}{\sum }}SINR_{l,k}}$, in the network above a threshold, which is specified; hence the range of IRSs positions that able to obtain this, can be determined. For simplifying the search space, i.e., decreasing the number of possible IRSs positions, we consider a system with 2 UEs and 2 IRSs. In this case, there are two available association patterns: firstly, IRS 1 and 2 associate with UE 1 and 2, respectively, while the second configuration is when IRS1 and IRS 2 associate with UE 2 and UE 1, respectively. The calculation is conducted in the spatial discretization space for potential IRSs and UEs positions as we will explain in detail in the Section 6.

## 5. Proposed NN Based Scheme for IRSs-UEs Association

In this section, we will describe the proposed neural network based IRSs-UEs association scheme in detail. This scheme is divided into two main stages, first estimating the angle of arrival (AoA) of each UE, and requesting to communicate through 150 GHz band, using MUSIC-based method. The second step is feeding a neural network with these estimated angles, so that the NN can determine a IRSs-UEs assignment matrix, i.e., associating each UE to certain IRS. At this point, a P-BF process is ready to be performed, within each IRS, to design the phase shifts of each IRS element. In the following two subsection, we will explain those two stages in more detail.

### 5.1. Estimating AoAs Using MUSIC Algorithm

The process begins when UEs request to establish a communication link; based on Wi-Fi band, AoAs of those UEs are estimated. Angle of arrival estimation can be considered as positioning information that is used to help in relaxing the complexity of different problems in communication systems. In our scenario, because of the availability of one AP in this indoor area, we adapt AoA estimation. However, if the location of UEs is provided using other services, e.g., GPS in outdoor, Wi-Fi in urban, or visible light communication in indoor environments, with high localization accuracy, it will be more efficient and accurate to use x-y-z coordinates of UEs in solving the IRSs-UEs association problem. To estimate AoA, we use the MUSIC-based approach due to its high accuracy for estimating AoAs, though other methods such as root-MUSIC [43] and ESPRIT [44] can be utilized. The concept of estimating AoA is briefly represented as follows:

The Wi-Fi AP has a uniform linear array (ULA) antenna, which consists of P isotropic antennas detached with a distance $d_{w}\leq \lambda_{w}/2$, where $\lambda_{w}$ refers to the wavelength of the plane waves received by the ULA. We assume that the ULA is hit by *V* narrowband plane waves from directions $\psi_{1},\;\psi_{2},\;\ldots .,\;\psi_{V}$ from the main response axis and $\pi /2\leq \psi_{1}\leq \;\psi_{2}\leq \ldots \leq \;\psi_{V}\leq -\pi /2$. Hence, at the time instance *q*, the received signal vector $x\left(q\right)\in {\mathbb{C}}^{P\times 1}$ can be written as:

(8) $$x\left(q\right)=\underset{i}{\sum }a\left(\psi_{i}\right)s_{i}\left(q\right)+n_{w}\left(q\right),$$

where $s_{i}\left(q\right)$ refers to the complex envelope of the *i*th signal, $n_{w}\left(q\right)\in {\mathbb{C}}^{P\times 1}$ is the vector of noise at the time instance q, and $a\left(\psi_{i}\right)$ is the vector of array response expressed as [45]:

(9) $$a\left(\psi_{i}\right)=\frac{1}{\sqrt{P}}{\left[e^{j\frac{2\pi }{\lambda }d_{w}sin\left(\psi \right)},\;\ldots ,\;e^{j\left(P-1\right)\frac{2\pi }{\lambda }d_{w}sin\left(\psi \right)}\right]}^{T}.$$

so, (8) can be expressed in matrix form as:

(10) $$x\left(q\right)=As\left(q\right)+n_{w}\left(q\right),$$

where $A=\left[a\left(\psi_{1}\right),\;a\left(\psi_{2}\right),\;\ldots ,\;a\left(\psi_{V}\right)\right]\in {\mathbb{C}}^{P\times V}$ and $s\left(q\right)={\left[s_{1}\left(q\right),\;s_{2}\left(q\right),\;\ldots ,\;s_{V}\left(q\right)\right]}^{T}\in {\mathbb{C}}^{V\times 1}$. MUSIC-based algorithm estimates AoA using covariance matrix of the received signal, which is expressed as:

(11) $$R=E\left[x\left(q\right)x{\left(q\right)}^{H}\right]=ASA^{H}+\sigma^{2}I_{P},$$

and where S refers to the covariance matrix of transmitted data given as $E\left[s\left(q\right)s{\left(q\right)}^{H}\right]$ and $I_{P}$ is the identity matrix with size P×P.

By taking Q snapshots, we take 50 snapshots in our work. A sample covariance matrix $\bar{R}\in {\mathbb{C}}^{P\times P}$ is evaluated as:

(12) $$\bar{R}=\frac{1}{N}\overset{K}{\underset{k=1}{\sum }}x\left(q\right)x{\left(q\right)}^{H},$$

From the eigenvalues of $\bar{R}$, we can obtain the signal and noise powers. Hence, pseudo-spectrum is calculated as:

(13) $$P\left(\psi \right)=\frac{1}{a{\left(\psi \right)}^{H}}U_{n_{w}}U_{n_{w}}{}^{H}a\left(\psi \right),$$

where $U_{n_{w}}\in {\mathbb{C}}^{P\times P-V}$ is the noise subspace. Thus, the estimation of AoA of UEs can be obtained for use in the following stage as an input for the neural network.

### 5.2. Neural Network for IRSs-UEs Association

**Figure 3:**
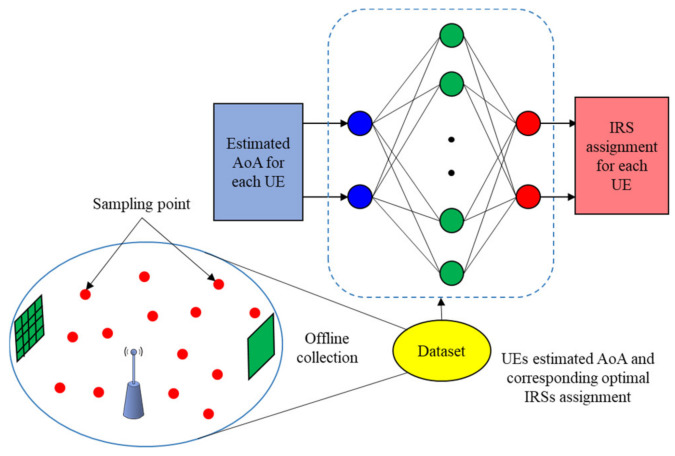
Proposed neural network for IRSs-UEs association.

Our pattern recognition NN for IRSs-UEs association is shown in Figure 3, where the estimated AoAs of UEs, obtained using the MUSIC algorithm, are considered as the input features for NN while the corresponding IRS assignments for each UE constitute the output of the network. We assume one hidden layer with B neurons; we will discuss the effect of changing the number of neurons on our NN performance on Section 6. The NN has been trained, validated, and tested with the collected dataset, which consists of $N_{s}$ samples. We study the effect of dataset size on the network accuracy in the Section 6. This dataset is generated by a raytracing model, and it obtains a high accuracy as we will discuss in the Section 6. To collect this dataset, we install our scenario, i.e., we deploy one AP and two IRSs in the indoor medium environment. Then, we uniformly distributed UEs in the area for 10,000 trials. Hence, the collected dataset, that used for NN model, contains the effect of UE location on the channel, e.g., path loss attenuation and multipath effect. After that, we use the model, that described in Section 3, to generate the SINR for each UE. Moreover, for each trial, the AoA of each UE is estimated, and exhaustive search is performed for determining best IRSs-UEs association patterns. We considered the values of these 10,000 trials as the dataset samples, where each sample contains the two UEs estimated AoA and the IRS index that they were associated with.

Here, for simplicity, we assume two inputs and two outputs network because the number of UEs is assumed to be two; in the future, extension by using other UEs information, e.g., positioning and required quality of services, with different number of UEs, i.e., multiuser scenarios, as inputs to NN can be proposed. The dataset samples are randomly divided between different sets, where 70% of the samples are used for training, while the other 30% of the samples are divided between validation and testing sets. The weights and biases are randomly initialized as small numbers and a scaled conjugate gradient algorithm is used to adjust them in the steepest descent direction using its default parameters. We also use a cross-entropy function to evaluate the network performance.

**Figure 4:**
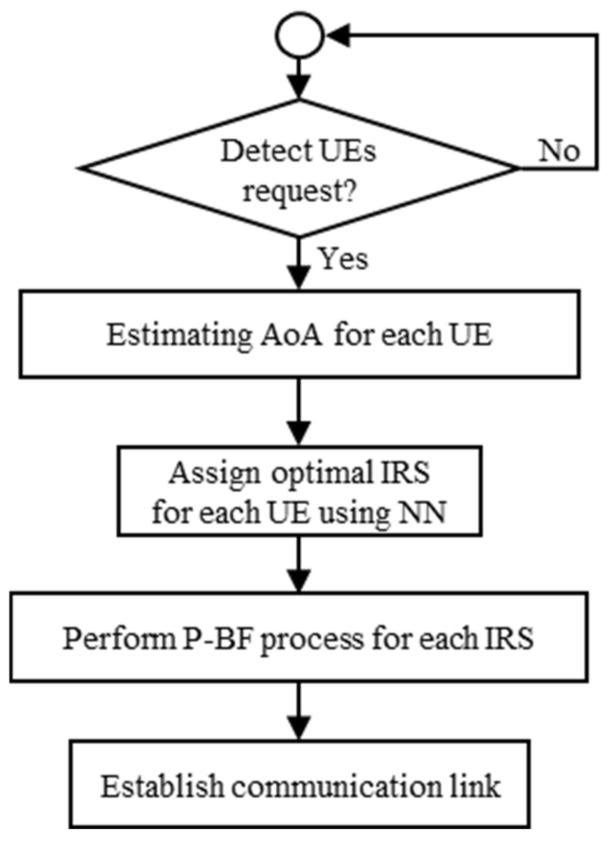
Flow chart of the joint NN based IRSs-UEs association and P-BF scheme.

Using NN for IRSs-UEs association can efficiently reduce the overall complexity in the network. Hence, we divide the joint assignment and P-BF problem to IRSs-UEs association, which is solved depending on the proposed NN previously discussed, and a P-BF problem within each IRS. The complete flow chart describing the joint IRSs-UEs association and P-BF scheme is presented in Figure 4. The scheme runs from the beginning of each time frame to establish the indirect AP-IRSs-UEs links for communication when direct AP-UE links are fully blocked. Specifically, when UEs need to communication over the 150 GHz band for high data rate application, UEs feedback their 5 GHz band signals to the AP using the broadcast channel. Hence, the AP can estimate the AoA of each UE using the MUSIC-based algorithm and forward these values to the input stage of the neural network. Based on the pre-trained NN, the AP can determine the corresponding IRS assignment for each UE, i.e., perform IRSs-UEs association. Then, it can direct its transmitted beams to the selected IRS. After that, for each IRS, the P-BF algorithm is performed once for designing the phase shift matrix of this IRS, which is assigned to certain UE. Consequently, we avoid performing the P-BF process for all possible IRSs-UEs configurations; in contrast, it is done once for single IRSs-UEs association pattern.

## 6. Simulation Results

In this section, firstly, optimal placement of two IRSs in the MIMO system for a beyond 5G network will be discussed, where we will compare our criterion in (7) with the methodology suggested in [27], as it can be considered as a reference method. Secondly, we will study the performance of the proposed NN based IRSs-UEs association scheme comparable to the conventional exhaustive search, iterative search and random based association schemes. Table 1 summarizes the common simulation parameters for both studies except if other values are mentioned in the context. At every trial, UEs are distributed uniformly, at height of 1 m, in any position in the studied indoor environment with dimensions 20 × 20 × 5 m^3^. The dual band AP, with 5 GHz and 150 GHz, is implemented at the center of the room, while IRSs pair can be placed anywhere on the top wall of the area. The simulation results are obtained after averaging on 100,000 Monte Carlo trials.

### 6.1. Effect of Optimal IRSs Placement in the Network

In regard to placing IRSs in an indoor 5G network, deploying IRSs depends on maximizing the minimum received power at UEs. Critical points are not considered, as we assume that each UE needs to be associated to the network and there is no interference signal that will affect its received signal. This is a non-practical scenario for downlink transmission in real networks, where more than one UE requests association to the network. For example, in a multi IRSs based system, the network can serve several users simultaneously using available indirect AP-IRSs-UEs links. Hence, mutual interference between these links will degrade the received signal at each UE. Moreover, even if we generalize this reference methodology to cope with multi IRSs scenarios by implementing IRSs based on maximizing the minimum UE SINR, the problem will not be resolved. This is because the reference strategy will only guarantee a certain level of SINR at UEs at the expense of wasting the opportunity to improve the total performance of the network. For studying the optimum placement of two IRSs in the network, we will search on all available space to implement the IRSs pair. It is just an example to implement two IRSs, as more than two can be used at the expense of increasing network complexity during communication in real time. To do this, we discretized the indoor area wall with a step of 0.1 m. For the possible IRSs positions, the calculation of the summation of UEs SINR for each trial is performed, after which we average over 100,000 Monte Carlo trials to handle all possible positions of UEs.

**Figure 5:**
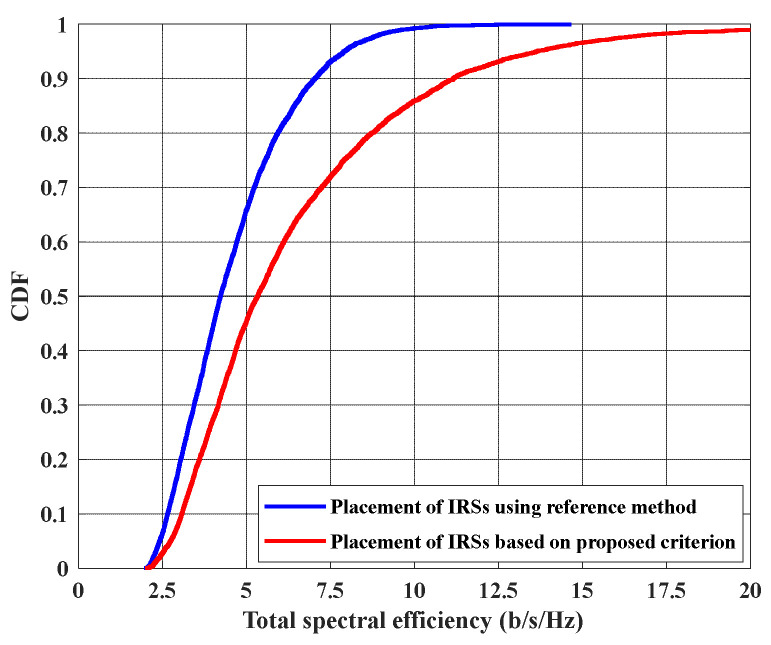
CDF of total spectral efficiency when placing the two IRSs using the proposed method, where IRSs positions are in (10, 0) and (10, 20), and using ref. [27] method, where IRSs positions will be in (0, 20) and (20, 20).

Figure 5 shows the cumulative distribution function (CDF) of total spectral efficiency, in the case of using the proposed criterion and reference strategy to place IRSs in the network. The total SE effects with the position of the two IRSs in the network, where the nearer IRS will generate higher mutual interference and farther IRSs will cause lower coverage. Hence, optimal placement of the two IRSs should be considered first. If the reference strategy is utilized, the two IRSs positions will be in (0, 20) and (20, 20) in an x-y coordinates system. This is because these two positions guarantee lower interference between UEs signals, thus giving maximum minimum SINR at UEs. Notice that, any adjacent corners, e.g., (20, 20) and (20, 0), are possible and we will obtain the same results. In contrast, our proposed criterion suggests placing the two IRSs in (10, 0) and (10, 20) as optimal positions. From Figure 5, it is clear that the proposed method is better than the reference one, where a higher SE can be obtained in the network by implementing IRSs based on the proposed principle. For instance, it can provide 5 bits/s/Hz total spectral efficiency for 55% of possible UEs positions, i.e., for 55% of the cases of different UEs positions in the area, while the reference method can obtain that for only 35% of UEs positions cases. The maximum total SE that can be achieved by the reference criterion is 10 bits/s/Hz. Meanwhile, the proposed methodology guarantees this SE for 14% of UEs position cases and can achieve 20 bits/s/Hz as a maximum total SE.

**Figure 6:**
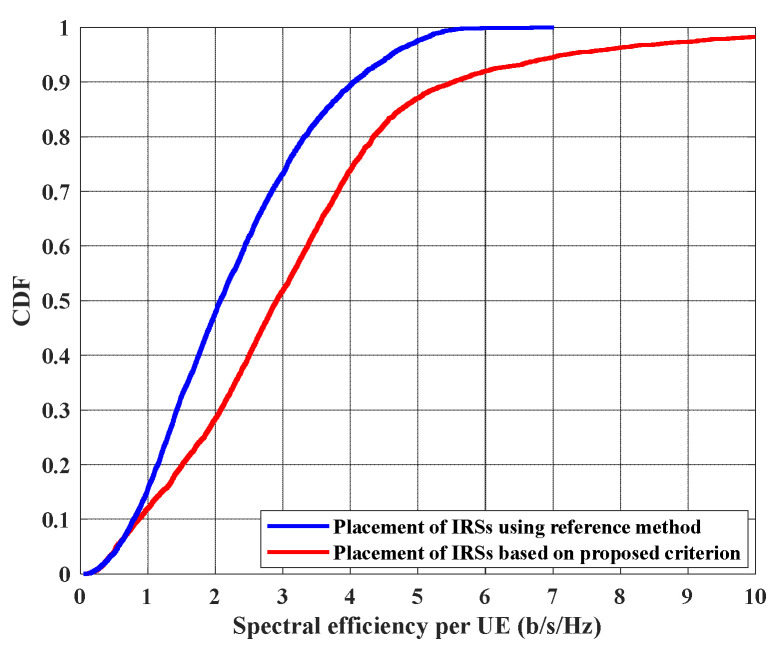
CDF of spectral efficiency per UE when placing the two IRSs using the proposed method, where IRSs positions are in (10, 0) and (10, 20), and using ref. [27] method, where IRSs positions will be in (0, 20) and (20, 20).

In Figure 6, the performance in terms of UEs point of view is studied where the CDF of spectral efficiency per UE is shown when both methodologies are adapted to deploy IRSs pair. This result proves that even each UE benefits from optimal placement of IRSs, i.e., the proposed criterion maximizes the SE for each UE not just increasing it for one UE at the expense of decreasing it for the other one. Again, the proposed criterion in (8) outperforms the reference methodology. For example, 50% of UEs can provide spectral efficiency larger than 2 and 3 bits/s/Hz, when the reference and proposed methods are used, respectively. Furthermore, some UEs, when they exist in certain positions, can obtain 10 bits/s/Hz as a maximum SE, if the proposed methodology is used, while they only achieve 5.5 bits/s/Hz as maximum SE, if the reference method is utilized.

**Figure 7:**
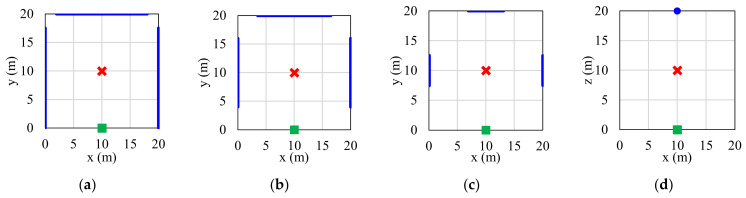
Optimal IRSs pair placement, with fixing first IRS, is marked with green square, in the middle of the wall at position (10, 0, 5), and study position of second IRS. The blue lines mark the possible positions for placing the second IRS to guarantee average total SINR larger than (**a**) 29 dBm, (**b**) 31 dBm, (**c**) 31 dBm, and (**d**) 35 dBm. AP is marked with red cross and its TX gain is 45 dBm.

In Figure 7, the placement of two IRSs is studied to guarantee achieving average total UEs SINR larger than a certain threshold. Hence, this will give more flexibility to deploy IRSs in the network. Firstly, we fix the first IRS in its optimum position, (10, 0, 5), represented by a green square, then change the position of the second IRS accordingly to examine all available positions. For each possible positions of IRS pair, the average total UEs SINR is calculated, then the value is compared with the specific threshold, and the procedure is repeated for next possible IRS pair positions till evaluating all available positions. For clarification purposes, we assume the AP transmitted gain equals to 45 dBm. The IRS pair positions that can provide average total UEs SINR above desired threshold are marked with blue line or dot as shown in Figure 7a–d. For instance, in Figure 7a, any position for a second IRS, where x=0 and $y\in \left[0,17.5\right]$ or $x\in \left[2,18\right]$ and y=20 or x=20 and $y\in \left[0,17.5\right]$, can guarantee average total SINRs larger than the threshold of 29 dBm. Then, if this threshold is increased to 31 dBm and 33 dBm, as shown in Figure 7b,c, narrower regions will be available to place the second IRS. Finally, selecting a threshold of 35 dBm, as shown in Figure 7d, leaves only one position for placing the second IRS, which is in the middle of the wall that is opposite to the wall where we deploy the first IRS. Thus, the optimum IRS pair positions will be (10, 0) and (10, 20). In case of increasing the threshold, no position will be available for the second IRS deployment, meanwhile decreasing the threshold, e.g., to 20 dBm, will make the entire walls suitable to place the second IRS.

### 6.2. Results of NN Based IRSs-UEs Association Scheme

**Figure 8:**
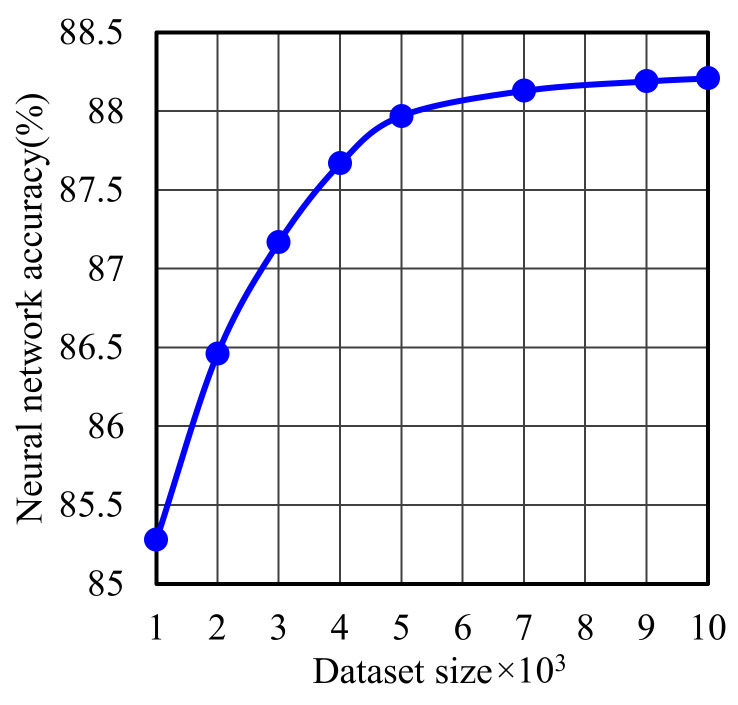
The accuracy of the network against the dataset size.

In Figure 8, we study the impact of dataset size on the accuracy of the network considering 10 units in NN hidden layer. One of the points that is still under question in artificial intelligence based schemes is the accuracy of the collected data for training the models. Hence, a large enough dataset, containing several samples to describe different channel randomness and changes, is needed to overcome the error in the data, e.g., errors in estimated UE AoA. From Figure 8, it is shown that the training accuracy increases with use of more samples in the dataset. However, to achieve balance between size and accuracy, we find that a 5000 sample is enough to train our scenario and obtain 88% accuracy.

**Figure 9:**
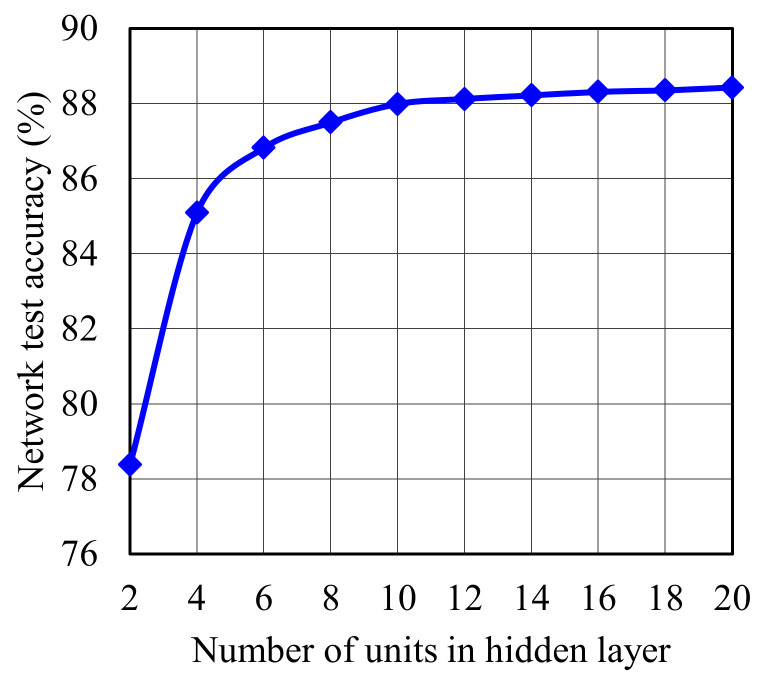
Network size against network accuracy.

In Figure 9, we evaluate the accuracy of our proposed simple NN model against the network size. It is obvious that increasing the number of hidden layer units enhances the accuracy of the NN, hence improving the overall system performance. However, this improvement will increase the complexity and as a result produce more delay, which is problematic for 5G networks. For this reason, in our NN, which is used for associating UEs to IRSs in the system, we use 10 bias units to balance between the system complexity and NN accuracy, where these ten units can obtain nearly 88% test accuracy for the network. This accuracy is acceptable given the simplicity of the network, the small size dataset, and inaccurate estimated angle of arrivals, which are used in the dataset. The generated delay from the network is low. Nevertheless, in the future, with more random and changeable scenarios, e.g., mobility cases, the required dataset size, the number of hidden layers, and its units should be reconsidered in the neural network design. Furthermore, adapting online learning to update the dataset is a promising direction to handle changes in the environment.

**Figure 10:**
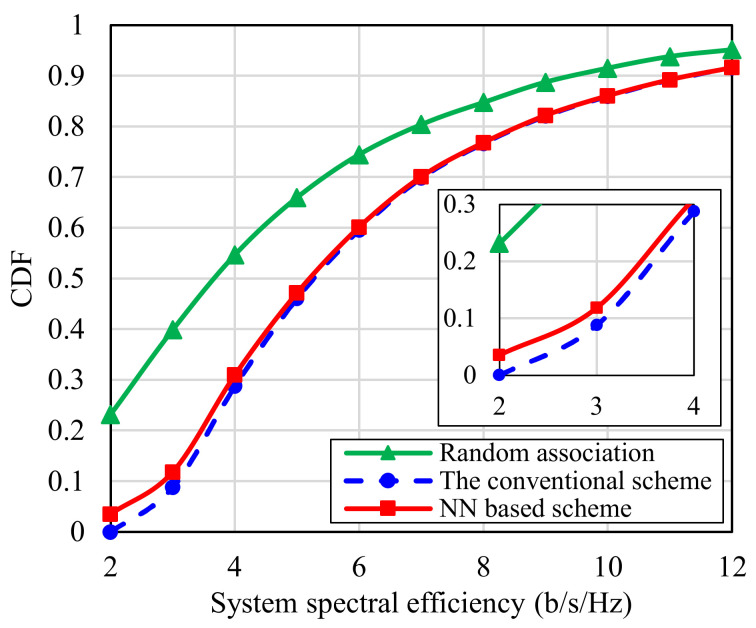
CDF of spectral efficiency using the proposed NN based scheme, the conventional exhaustive search based scheme, and random association based scheme.

Figure 10 presents the CDF of total spectral efficiency (SE) when the proposed NN based IRSs-UEs association scheme is used, comparing it with the SE obtained with the conventional exhaustive search based association scheme and the random association based scheme. From this figure, we can see that the performance of the SE with our proposed scheme is nearly identical to the optimum performance, that achieved with the higher power and time consuming conventional scheme. However, it is to be noticed that a small difference exists due to the accuracy of NN. Moreover, the proposed scheme guarantees better SE comparable to the random association based scheme though it needs the same complexity. For example, the probability to obtain SE larger than 6 b/s/Hz is 0.399, 0.404, and 0.2552, if the proposed scheme, the conventional scheme, and the random association based scheme are adopted, respectively.

**Figure 11:**
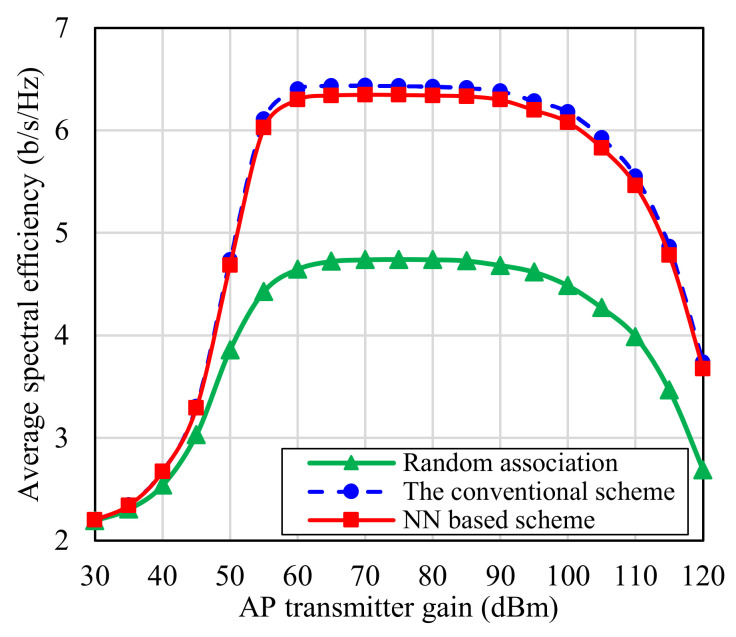
The total average spectral efficiency (SE) against AP transmitter gain.

Another important factor worth study is the effect of the AP antenna gains on the overall performance of the network. Figure 11 illustrates this point, where the average spectral efficiency of the system is studied against the transmitted AP antenna gain, while setting the UE antenna gain on 20 dBm. The SE increases with rising the TX antenna gain, between 30 dBm and 60 dBm, until reaching to a saturation point, where the SE obtained by the proposed and conventional schemes increases from 2.2 bits/s/Hz to nearly the triple, 6.3 bits/s/Hz. After reaching the maximum received power value, the TX antenna gain has no effect [42], where both the received and interference power decreases nearly with the same level. Moreover, both of them are still very large in comparison to noise level (−70 dBm). Hence, the SINR and the SE saturate between 60 dBm and 90 dBm TX antenna gains. Then, after 90 dBm gain, the received power will decrease more and the noise and interference powers will begin to dominantly contribute, where a −70 dBm noise power will have to be considered. Hence, the SE of the system dramatically decreases. Moreover, from Figure 11, it is proven that the proposed NN based IRSs-UEs association scheme works well comparable to a random association based scheme, whatever the gain value used by TX antenna. In addition, the proposed scheme performance is very near to the performance of the conventional scheme. For example, an average SE of 6.29 bit/s/Hz is obtained, at 60 dBm antenna gain, using the proposed scheme, which is far above the SE obtained by the conventional scheme with only 0.11 bits/s/Hz.

**Figure 12:**
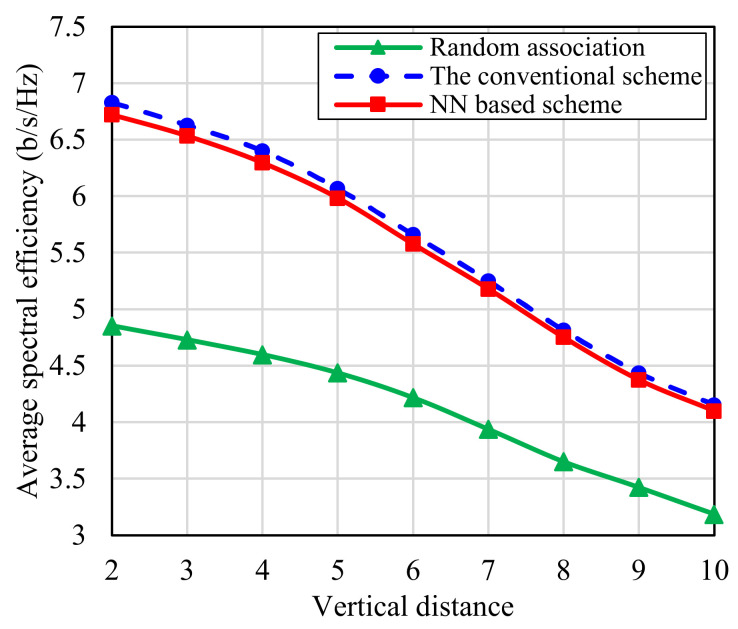
The average system spectral efficiency against vertical distance between IRSs plane and UEs plane.

In Figure 12, the impact of vertical distance between IRSs and UEs on the performance of the network is studied. This is done by changing the study area height from 3 m to 11 m, while fixing UE height at 1 m from the ground. As expected, increasing the vertical distance between IRSs and UEs decreases the average spectral efficiency due to the decline of the SINR. For example, the average spectral efficiency of the proposed scheme decreases from 6.7 bits/s/Hz to 5.57 bits/s/Hz by increasing the vertical distance from 2 m to 6 m, respectively. However, the proposed NN based scheme is still robust against changes in vertical distance because the used learning features are the estimated azimuth angles which are not affected by the vertical distance. Furthermore, the model still obtains the same performance as the conventional one even with the increase of the vertical distance. Figure 10, Figure 11 and Figure 12 show that the proposed NN based scheme can obtain suboptimum performance with much lower complexity comparable the optimum performance exhaustive search based scheme.

Finally, Table 2 shows the complexity of the proposed scheme for establishing AP-IRSs-UEs links comparable to the exhaustive search and iterative switching based schemes. The proposed scheme uses NN for IRSs-UEs association, then performs P-BF as explained in Section 5, while the conventional scheme searches on all possible IRSs-UEs patterns, which equal to $K!/\left(K-L\right)!$ patterns, and within each pattern, P-BF is performed for each IRS. Furthermore, we consider an iterative switching based scheme, that had been proposed in [28], for comparison purposes, after adapting it to our scenario. Assuming 1 AP, 2 UEs, and 2 IRSs, the total searching complexity of this scheme will be $2C_{P-BF}S$, where $C_{P-BF}$ is the complexity of performing P-BF in one IRS, and S is the number of times that IRSs-UEs association matrix is updated. Notice that this scheme firstly initializes the association matrix randomly. With this scenario, *S* will be equal to 0 or 1, hence the total computational complexity will be on the average 1155. In our scenario, with medium area, it is logical to install just two IRSs, because implementing more IRSs will increase the complexity to unnoticeably enhance the performance without much effect. The main merit of our proposed scheme is that it determines a best association pattern without searching or updating an association matrix, hence greatly reducing the complexity. The required complexity by the proposed scheme is only 50% and 66% of the complexity needed by the conventional scheme, which obtains the optimum performance, and the iterative switching based scheme, respectively. This reduction in operations reflects the decrease in time and power required for establishing AP-IRSs-UEs links comparable to the conventional scheme. Furthermore, updating association pattern until a convergence point in an iterative based scheme, which achieves suboptimum performance, needs feedback signaling and consumes power at every iteration, which is inefficient.

**Table 2:** Complexity and average spectral efficiency of the proposed NN based scheme comparable to the conventional scheme and iterative switching based scheme.

Metric	The Conventional Scheme	Iterative Switching Based Scheme [28]	The Proposed NN Based Scheme
Computational complexity	1540	1150	770
Average SE (bits/s/Hz)	6.40	6.38	6.29

If a multiuser scenario is considered in a MIMO system, the number of association patterns will increase, and hence the complexity of exhaustive and iterative search based schemes will largely increase. In contrast, with a modification on our machine learning model, an acceptable performance with low complexity can be achieved. Moreover, mmWave and THz channels are highly dynamic in time and affected by the environmental changes, e.g., moving humans, temperature, and humidity changes. In the future, a more reliable model can be considered, where the effect of changes in the environment, e.g., dynamic human blockage, can be handled, e.g., based on predicting blockage using works proposed in [11,46]. Moreover, instead of retraining the network when environment changes, a reinforcement learning (RL) based scheme can be proposed, where the network can learn and interpret the environmental behavior to achieve better performance. Nevertheless, the proposed NN based IRSs-UEs association scheme in our manuscript is efficient in stationary scenarios, e.g., 4K and 8K video streaming, histogram applications, with tactile internet.

## 7. Conclusions

In this paper, the IRSs-UEs association and optimal IRSs deployment problems in multi IRSs aided MIMO system are studied considering the impact of mutual interference between IRSs-UEs virtual links. First, we study the optimal placement of pair of IRSs and the effect of their positions on the performance of the network. We propose a deployment criterion based on the interference between IRSs-UEs links, where this strategy aims to maximize average total UEs SINR. This strategy guarantees better performance in terms of system SE and SE per UE comparable to the reference method. Secondly, a novel neural network based IRSs-UEs association scheme is proposed, where the estimated angle of arrivals of UEs, obtained using the accurate MUSIC algorithm, are used as an input features for NN. This association scheme reduces the complexity of the system, because it determines the best IRSs-UEs assignment pattern directly instead of searching all possible configurations to find out the optimum one, as the conventional scheme does. For instance, by deploying two IRSs and two UEs in the network, the complexity is reduced to 50% compared to the conventional scheme while maintaining nearly similar performance in terms of system spectral efficiency, where only 0.11 bits/s/Hz in SE is reduced. The proposed NN based scheme also preserves the performance near to the conventional approach performance when the transmitted AP gain or the vertical distance between IRSs and UEs are increased, as it is only based on the angles between the AP and UEs. This work can be extended in the future by considering other contextual information, e.g., UEs position. Moreover, deploying multi IRSs in a multiuser MIMO system will be studied. Furthermore, a reinforcement learning (RL) based scheme can be proposed, where the model can learn from the environment to achieve better performance.

## Figures and Tables

**Table 1:** Simulation parameters.

Parameter	Value
AP transmitted power, *P_t_*	1 watt
Number of AP antennas, *M*	2
AP transmitted antenna gain, *G_t_*	60 dBm
UE received antenna gain, *G_r_*	20 dBm
Height of AP, IRSs, UEs	5 m, 5 m, 1 m
Operating frequency	150 GHz
Number of IRS reflecting elements	32
Common reflection amplitude, |*R*|	0.9
Number of dataset samples	5000
